# Review of Impact of COVID-19 on Maternal, Neonatal Outcomes, and Placental Changes

**DOI:** 10.7759/cureus.28631

**Published:** 2022-08-31

**Authors:** Resham Tanna, Henry J. Nava Dugarte, Sowjanya Kurakula, Vandana Muralidharan, Arghadip Das, Sri Padma Ravali Kanigalpula, Ileana Elita Mendez, Munaza Afaq, Radhika Bassi, Kinjal Shah, Zainab Saddiq

**Affiliations:** 1 Obstetrics and Gynaecology, Spartan Health Sciences University, Vieux Fort, LCA; 2 Obstetrics and Gynaecology, Rejuvenating Fertility Centre, New York, USA; 3 Obstetrics and Gynecology, Mamta Institute of Medical Sciences, Khammam, IND; 4 Obstetrics and Gynecology, Sekgoma Memorial Hospital, Serowe, BWA; 5 Obstetrics and Gynecology, Nyangabgwe Referral Hospital, Francistown, BWA; 6 Obstetrics and Gynecology, Gandhi Medical College, Musheerabad, IND; 7 Obstetrics and Gynaecology, Lifeline Medical Associates, New Jersey, USA; 8 Internal Medicine, Nilratan Sircar Medical College and Hospital, Kolkata, IND; 9 Obstetrics and Gynaecology, Ascension Health via Christi Hospital, Manhattan, USA; 10 Medical Sciences Department, Universidad Autonoma de Centro America (UACA), San José, CRI; 11 Obstetrics and Gynaecology, Government Medical College Srinagar, Srinagar, IND; 12 Obstetrics and Gynaecology, Ross University School of Medicine, Bridgetown, BRB; 13 Health Administration, Edward J. Bloustein School of Planning and Public Policy, New Jersey, USA; 14 Obstetrics and Gynecology, Annotto Bay Hospital, St.Mary, JAM

**Keywords:** placental changes, neonatal outcomes, maternal outcomes, pregnancy, covid-19

## Abstract

Coronavirus disease (COVID-19), caused by SARS-CoV-2, is a disease that has caused a global impact. COVID-19 is transmitted through airborne droplets, respiratory secretions, and direct contact. The pandemic has affected individuals of different ages, and studying the impact of COVID-19 on maternal and newborn outcomes is critical. In this review, we highlight the impact of COVID-19 infection in pregnancy and its repercussion in the maternal-fetal binomial. Physiological changes that occur during pregnancy have significant effects on the immune system, cardiopulmonary system, and coagulation, and these changes can result in an altered response to COVID-19 infection. The symptoms, risk factors, and maternal health consequences of COVID-19 were discussed. In addition, the impact of newborns born to mothers with COVID-19 was reviewed. Finally, placental changes and vertical transmission of COVID-19 during pregnancy were also discussed in this review.

## Introduction and background

The first outbreak of coronavirus disease (COVID-19) caused by severe acute respiratory syndrome coronavirus-2 (SARS-CoV-2) was reported in December 2019 in Wuhan, China. It was declared a global pandemic in March 2020 with a total of 505,817,953 confirmed cases and 6,213,876 deaths reported by the World Health Organization (WHO) as of April 2022 [1]. COVID-19 illness is transmitted through airborne droplets, respiratory secretions, and direct contact [2]. While the symptoms related to COVID-19 disease are primarily respiratory, the disease has been reported to have multisystemic effects. COVID-19 illness symptoms can be asymptomatic, mild, moderate, severe, or critical [3,4,5]. Fever, cough, dyspnea, and myalgia were the most common mild symptoms [6-10]. The most common imaging findings were ground-glass opacities, and bilateral infiltrates, and the most common laboratory findings were leucopenia, thrombocytopenia, and elevated CRP [6,10]. Less common symptoms were nausea, vomiting, and diarrhea [11]. 

The Centers for Disease Control and Prevention (CDC) defines critical illness in a person with COVID-19 as a person who may require hospitalization, admission to an intensive care unit, and mechanical ventilation [3]. In addition, pregnancy is also listed as an underlying medical condition that increases the risk of contracting COVID-19 illness [3]. Multiple studies have found that pregnant women with severe or critical COVID-19 disease require oxygen, mechanical ventilation, and ICU admissions [4,12-15]. A variety of studies have shown that maternal mortality rates range from 0.3%, 0.74%, 1.37%, 1.60%, one in 80, and seven in nine women with critical COVID-19 illness in pregnancy [5,13,16-19]. Conflicting results were reported in a study reporting that there is no significant correlation between COVID-19 pregnancy and mortality [20].

The effects of the COVID-19 pandemic are not limited to its associated morbidity and mortality, it has also led to social and economic disruption around the world and has through ripple effects impacted the healthcare system in many ways, affecting the quality of patient care. The maternal health care system which includes prenatal care, obstetrics, and postpartum care, has also undergone major changes in its structure [21]. During social distancing measures in some parts of the UK, women were provided with blood pressure monitors and urine dipsticks to do prenatal screening themselves [22]. Kotlar et al., reported that fewer prenatal visits, a strained healthcare infrastructure, and potentially harmful policies were implemented with limited evidence during the pandemic [23]. On the other hand, telemedicine has evolved and has been extremely useful for prenatal consultations, although underutilized in low-income countries [22].

Pregnancy is generally considered to be a high-risk condition associated with infectious diseases because the immunological changes of pregnancy can increase susceptibility to pathogens and associated complications [11]. Pregnant women are listed as a high-risk population for COVID-19 disease [24]. Physiological changes that occur during pregnancy have significant effects on the immune system, cardiopulmonary system, and coagulation, and these changes can result in an altered response to COVID-19 infection [25]. 

The impact of the disease on maternal and fetal outcomes has been questioned since the pandemic began. In this review, we explore the latest research on the maternal and neonatal consequences of COVID-19. We also highlight the peculiar placental changes that occur as a result of this infection.

## Review

Impact of COVID-19 on maternal outcomes

According to the WHO, COVID-19 infection is more severe in pregnant women than in non-pregnant women [26]. Some ethnic groups, such as Hispanics, Blacks, and Asians, had higher rates of moderate and severe COVID-19 illness [3,27]. Additionally, the CDC found that pregnant women of the same ethnic group are at increased risk for COVID-19 illness due to health inequalities they face [28]. Son et al. however, found no differences in pregnancy-related outcomes between pre- and during-pandemic deliveries and those testing positive or negative for COVID-19 disease in geographically diverse US cohorts [29].

Several studies have compared pregnant women who tested positive for COVID-19 disease with those who tested negative [12,13,20,27,29,30]. Pregnant women with COVID-19 disease were more likely to develop serious outcomes if they had comorbidities such as chronic obstructive pulmonary disease (COPD), asthma, type 1 and type 2 diabetes, and obesity [4,10,12,17,27,31,32]. A variety of studies have identified maternal age > 40, hypertension, and autoimmune disorders as additional risk factors for contracting SARS-CoV-2 infection in pregnancy [3-5,12,27,31,33]. Wei et al. found that severe COVID-19 disease is strongly associated with preeclampsia [30]. There were high rates of preeclampsia and chronic hypertension, preterm birth, and cesarean births in pregnant patients with a critical COVID-19 illness [4,8,10,13,14,27,34]. An increased likelihood of placental abruption due to SARS-CoV-2 infection was noted by Marta and colleagues [35]. In one study, fetal growth restriction and premature prelabor rupture of membranes (PPROM) were observed in 11.7% and 20.7%, respectively [36]. It is however reported that the clinical course and severity of COVID-19 pneumonia and severe COVID-19 illness during pregnancy are similar to those of non-pregnant women [33,34,37,38]. In addition, various organ system involvement in COVID-19 disease in obstetric populations was discussed by Syeda et al., which is represented in Table 1 [11]. It is however important to note that some of these findings can also be due to direct obstetric complications [11].

**Table 1:** Impact of COVID-19 in pregnancy on various maternal organ systems. Original figure drawn by authors. Abbreviations: HELLP = Hemolysis, Elevated Liver enzymes, Low Platelets

Impact of COVID-19 on various maternal organ systems				
Cardiac	Pulmonary	Renal	Hepatic	Hematological
Cardiomyopathy	Silent hypoxia	Acute Kidney Injury	Elevated transaminases	Neutrophilia
	Risk of pulmonary edema due to combined acute kidney injury and pulmonary injury		Findings mimicking HELLP syndrome and severe preeclampsia	Lymphopenia
	Mixed and complete consolidations on chest imaging			

A notable increase in mental illness in the form of self-harm, depression, and anxiety have also been observed in pregnant women with COVID-19 illness [16,39,40]. Domestic violence increased among pregnant women during the pandemic, they had lower incomes than men, and working mothers had to deal with increased childcare needs [22,23]. One study had reported lower vitamin D levels in COVID-19 affected pregnancies compared to healthy pregnancies [41].

Impact of COVID-19 on neonatal outcomes

The impact of maternal SARS-CoV-2 infection on the fetus and subsequently the neonate remains poorly understood [42]. The increasing number of reports of neonates born to women with COVID-19 showing signs of early-onset infection is suggestive of a scenario of transplacental transmission of SARS-CoV-2 [43-45]. However, vertical transmission of the virus remains controversial as clinical and laboratory evidence considers it a rare event [9,44,46-51]. In particular, a cause-and-effect relationship between viral infection and the adverse neonatal outcome has not been formally established, since other factors causing postpartum complications cannot be ruled out [43,49]. Therefore, it remains uncertain whether early neonatal SARS-CoV-2 infection occurs in utero, during birth, or shortly after birth [52].

Pregnant women with COVID-19 have high rates of spontaneous preterm birth, with studies reporting rates of 21% - 40% spontaneous preterm birth in their cohorts, which is two to four times the average rate of 10% [36,53-55]. Approximately 3% of pregnancies in women with COVID-19 end in stillbirth, with the usual rate being < 1% in pregnant patients without COVID-19 illness [56,57]. Studies have shown that placental damage from SARS-CoV-2 infection is sufficient to cause severe morbidity and even mortality in newborns [51,58,59]. This remains controversial as reports show that stillborn newborns and their placenta are negative for SARS-CoV-2 infection despite extensive placental damage and funisitis [51,58,59]. In addition, the absence of symptoms in the infected mother does not rule out the death of the newborn [51,58,59]. A case study by Poisson et al. reported a SARS-CoV-2-positive asymptomatic pregnant woman at 32 weeks gestation, went on to have a still birth after 35 weeks gestation. The stillborn baby showed no abnormalities, but 75% of the placenta showed extensive fetal vascular insufficiency and parenchymal infarction [60]. In another study, two newborns of symptomatic COVID-19 women tested positive for nasopharyngeal swabs shortly after birth and seven days after birth, but neither developed infection-related symptoms [61]. The placenta in both cases was positive for SARS-CoV-2 by in situ hybridization and Reverse Transcriptase-Polymerase Chain Reaction (RT-PCR) analysis [61]. Facchetti et al. reported a case of a SARS-CoV-2-positive pregnant woman with pneumonia and severe thrombocytopenia requiring induction of labor. The newborn was positive for the presence of the virus and developed pneumonia and severe shortness of breath 24 hours after birth [62]. In summary, their observations provide evidence of the development of intrauterine vertical transmission of SARS-CoV-2 infection and have infrequent clinical effects on newborns [62].

In a multinational cohort study, the risk of major neonatal complications, including a stay in the neonatal intensive care unit (NICU) of at least seven days, as well as the summary index of major neonatal morbidity and its individual components, were also significantly higher in the group of women diagnosed with COVID-19 disease [13]. The increased neonatal risk persisted after adjustment for earlier preterm births and preterm births in the index pregnancy; therefore, a direct impact of COVID-19 on the newborn is likely. They found that 12.1% of newborns born to test-positive women also tested positive, a higher number than in a recent systematic review. Reassuringly, since SARS-CoV-2 has not been isolated from human milk, breastfeeding was not associated with an increase in the rate of test-positive newborns [63]. Many studies have not detected the SARS-CoV-2 virus in breast milk and have also confirmed that SARS-CoV-2 antibodies are transmitted through breast milk [47,64,65]. The WHO states that breastfeeding is the optimal form of nutrition for babies and recommends that women infected with COVID-19 breastfeed their babies if possible [66].

Placental changes due to COVID-19 infection

Pregnancy is a time of immune regulation that allows the fetus to develop in the womb while protecting the mother from infection. The state of placental immune protection for the fetus in a normal pregnancy could potentially have an adverse effect on pregnancy affected by COVID-19 [67].

During pregnancy, many fetal and neonatal pathologies are associated with cross-placental transmission of viruses such as rubella, varicella, cytomegalovirus, dengue, and zika, among others. Evidence of fetal and maternal vascular malperfusion, as well as evidence of inflammation in the placenta following maternal SARS-CoV-1 infection, have been reported [68].

Histopathological examination of placental tissue can provide important information about the condition of the mother and fetus [51]. Laresgoiti-Servitje et al. 2021, reported that the placentas of women infected with SARS-CoV-2 had a higher rate of fibrinoid deposition, a clinical feature of maternal vascular malperfusion (MVM), when compared with controls [69]. Additionally, it was noted by Shanes and Col., that relative to controls, COVID-19 placentas showed an increased prevalence of decidual arteriopathy and other features of MVM, a pattern of placental injury that reflects abnormalities in oxygenation within the intervillous space associated with adverse perinatal outcomes [51].

The angiotensin-converting enzyme 2 (ACE2) and the transmembrane protease serine 2 (TMPRSS2) are SARS-CoV-2 specific entry mediators that are highly expressed in the human placenta in early pregnancy, but their expression decreases significantly as the pregnancy progresses [70]. SARS-CoV-2 infection present in the maternal circulation has the potential to invade the maternal blood-soaked syncytiotrophoblast and infect the placenta via ACE2 binding. The higher levels of ACE2 mRNA in the placenta at earlier stages of pregnancy raise the possibility of higher susceptibility to SARS-CoV-2 infection in the placenta in the first trimester. It has been demonstrated that there is no evidence of vertical transmission when the infection manifests itself in the third trimester of pregnancy [9].

Pathological studies have shown that syncytiotrophoblasts are commonly infected with SARS-CoV-2, but fetuses are not always infected [71]. These findings suggest the presence of a placental barrier [71]. Facchettia et al., reported for the first time that SARS-CoV-2 S and N proteins were highly expressed in the placenta of a pregnant COVID-19 woman whose newborn tested positive for viral RNA and developed COVID-19 respiratory symptoms soon after birth [62]. The report revealed SARS-CoV-2 virus products and/or particles in villous syncytiotrophoblast, endothelial cells, fibroblasts, and maternal macrophages that contribute to inflammatory infiltration, as well as Hoffbauer cells. Surprisingly, particles morphologically consistent with the coronavirus localized into the fetal circulating mononuclear cells [62].

It is important to mention that other studies were found with no evidence of placental infection or vertical transmission of SARS-CoV-2 [72,73]. Such as that presented by Levitan, where after evaluation of placentas from 65 polymerase chain reaction women, proven SARS-CoV-2 infection and histological evaluation indicated that there is no characteristic histopathology in most placentas [72]. The same results were obtained from Smithgall in the study of 51 placentas of SARS-CoV-2 positive third-trimester mothers, where found non-specific histomorphologic changes that point to indicated maternal/fetal vascular malperfusion. All newborns tested negative for SARS-CoV-2 infection and all mothers recovered clinically [73]. This could be related to the fact that the transplacental transfer of anti-SARS-CoV-2 antibodies was inefficient and factors such as lack of viremia, reduced coexpression, and colocalization of placental angiotensin-converting enzyme 2 and transmembrane serine protease 2 as protection could serve mechanisms against vertical transmission [74].

Interpretation of placental changes and vertical transmission requires caution as there may be other undefined mechanisms linking obstetric outcomes of SARS-CoV-2 under normal and pathologic conditions.

## Conclusions

COVID-19 disease is an evolving disease with varying reports of a significant impact of severe COVID-19 illness on maternal outcomes. Various studies have shown conflicting results on the neonatal consequences of COVID-19 infection. The concept of placental changes and vertical transmission also remains inconclusive. Therefore, it is important to continuously investigate the impact of COVID-19 disease on maternal and neonatal outcomes, and its effect on vertical transmission and placental changes. 
